# Draft Genome Sequence of *Bacillus clausii* UBBC07, a Spore-Forming Probiotic Strain

**DOI:** 10.1128/genomeA.00235-16

**Published:** 2016-04-21

**Authors:** Aditya Upadrasta, Swetha Pitta, Ratna Sudha Madempudi

**Affiliations:** Centre for Research and Development, Unique Biotech Limited, Hyderabad, India

## Abstract

*Bacillus clausii* UBBC07 is a safe endospore-forming strain, characterized for defined therapeutic effects. The finished draft whole-genome sequence is presented here to scan its genetic constitution for its expanded use as a probiotic in various health sectors.

## GENOME ANNOUNCEMENT

The advantage of endospore formers over *Lactobacillus* species is their long shelf-life and high viability under hostile conditions. Very few members in the *Bacillus* group are recognized as safe for use in cultures; hence, only a few *Bacillus* strains are available as commercial preparations for application with humans, animals, and plants (1, 2). *Bacillus clausii* UBBC07 is a safe spore-forming probiotic, which has shown therapeutic effect in patients suffering from acute diarrhea (3). Furthermore, *in vitro* analyses revealed that *B. clausii* UBBC07 can grow at high salt concentrations, tolerate bile acids, survive under gastric conditions, and adhere to epithelial cells (our unpublished data).

Here, we report the draft whole-genome sequence of *B. clausii* UBBC07 in order to understand the molecular basis of probiosis and its safe use as a probiotic for human and animal health care. To the best of our knowledge, this is the first published genome sequence of a commercially available *B. clausii* probiotic strain.

Whole-genome sequencing was performed using the Illumina MiSeq platform (Institute of Microbial Technology [IMTECH], Chandigarh, India). The paired-end libraries were prepared using the Nextera XT sample preparation kit, with dual indexing adaptors. A total of 1,010,205,977 reads were obtained, providing 241× depth of genome coverage. Among them, 4,197,324 Illumina reads were *de novo* assembled using CLC Genomics Workbench version 7.5. The assembled genome sequence was annotated by the RAST annotation pipeline Web server (4, 5) and by NCBI Prokaryotic Genome Annotation Pipeline (PGAP) version 2.10 (http://www.ncbi.nlm.nih.gov/genome/annotation_prok/). rRNAs and tRNAs were annotated using RNAmmer (6) and tRNAscan-SE (7), respectively. The clustered regularly interspaced short palindromic repeat (CRISPR) gene clusters were identified by using CRISPRFinder (8). The genome analysis was performed using the Artemis genome viewer (9).

The draft genome sequence assembled into 61 contigs composed of 4,197,324 bp, with an average G+C content of 44.6%. RAST annotation revealed a total of 4,361 coding sequences (CDSs), including 8 rRNAs, and 75 tRNAs, with a coding percentage of 87.2% and 1.03 gene density per kilobase, which are in line with the *B. clausii* KSM-K16 genome. The genome consists of CRISPR cassettes, possibly involved in defense machinery toward foreign genetic elements and a putative bacteriocin operon.

The RAST SEED metabolic analysis revealed 2,955 functional genes (475 subsystems), in which the highest number accounted for central carbohydrate metabolism (576 genes, including xylose, sucrose, maltose, and maltodextrin and chitin utilization), amino acids and derivatives (423 genes), cofactors and vitamins (217 genes), protein metabolism (192 genes), RNA metabolism (162 genes), sporulation and dormancy (101 genes), and stress response (104 genes). Furthermore, the genome contains determinants involved in adhesion, i.e., fibronectin, an LPXTG motif, mucus binding, and bacillibactin siderophore formation. In addition to that, this strain did not harbor any deleterious genes that encode enterotoxins and hemolysins.

Generation of the complete genetic makeup of *B. clausii* UBBC07 has revealed many beneficial probiotic traits that contribute to the safety of the strain for its use in a wide range of health-promoting applications.

### Nucleotide sequence accession numbers.

This whole-genome shotgun project has been deposited at DDBJ/ENA/GenBank under the accession no. LATY00000000. The version described in this paper is version LATY01000000.
